#  Correction

**DOI:** 10.1111/cas.15680

**Published:** 2023-01-24

In an article^1^ titled “Long non‐coding RNA UCA1 contributes to the progression of oral squamous cell carcinoma by regulating the WNT/β‐catenin signaling pathway” by Yong‐Tao, Yang; Yu‐Fan, Wang; Ju‐Yi, Lai; Shi‐Yue, Shen; Feng, Wang; Jie, Kong; Wei, Zhang; Hong‐Yu, Yang, there were errors in Figures 2, 4 and 5.

The revised Figure 2(A) is shown below:

**Figure 2:**
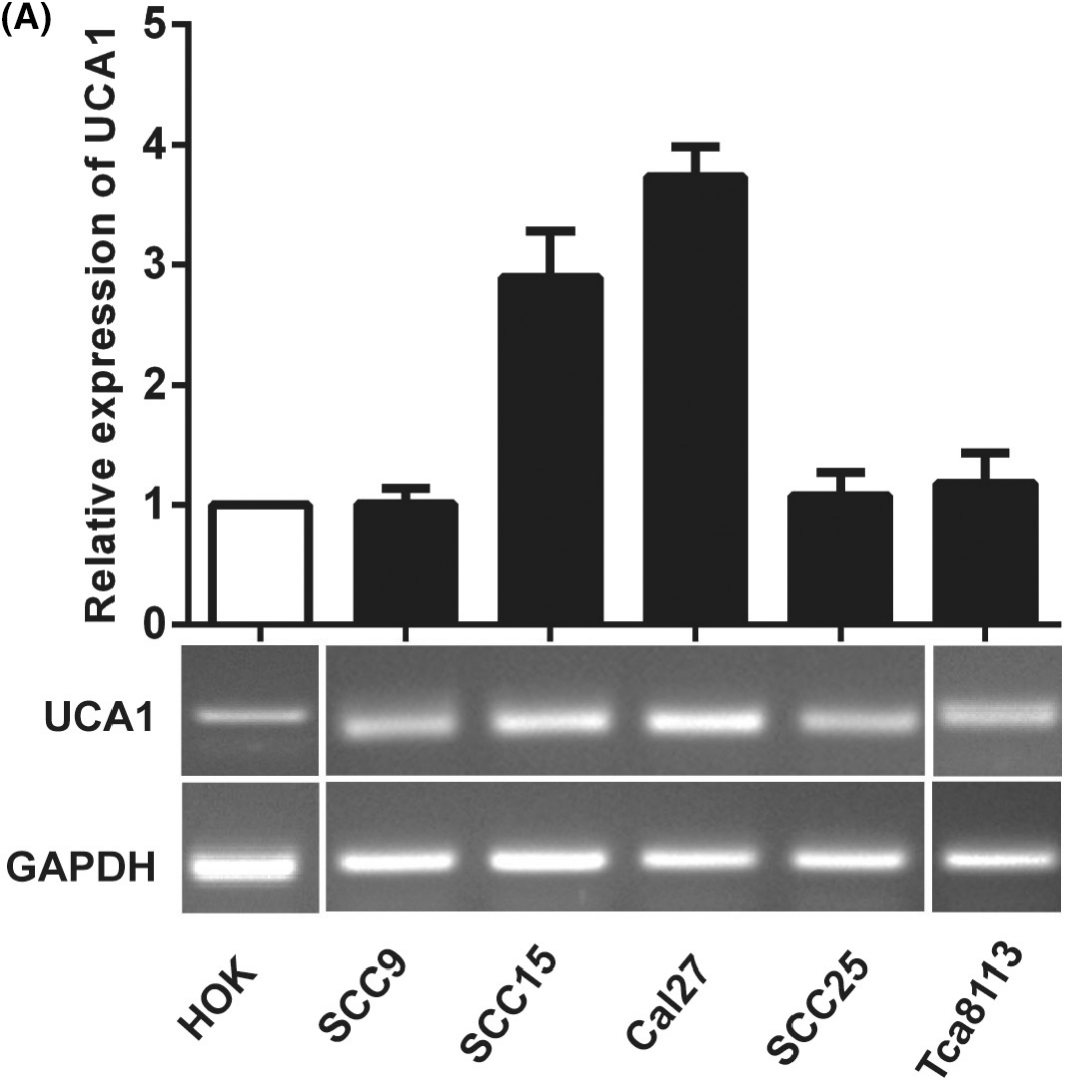


The revised Figure 4 is shown below:

**Figure 4:**
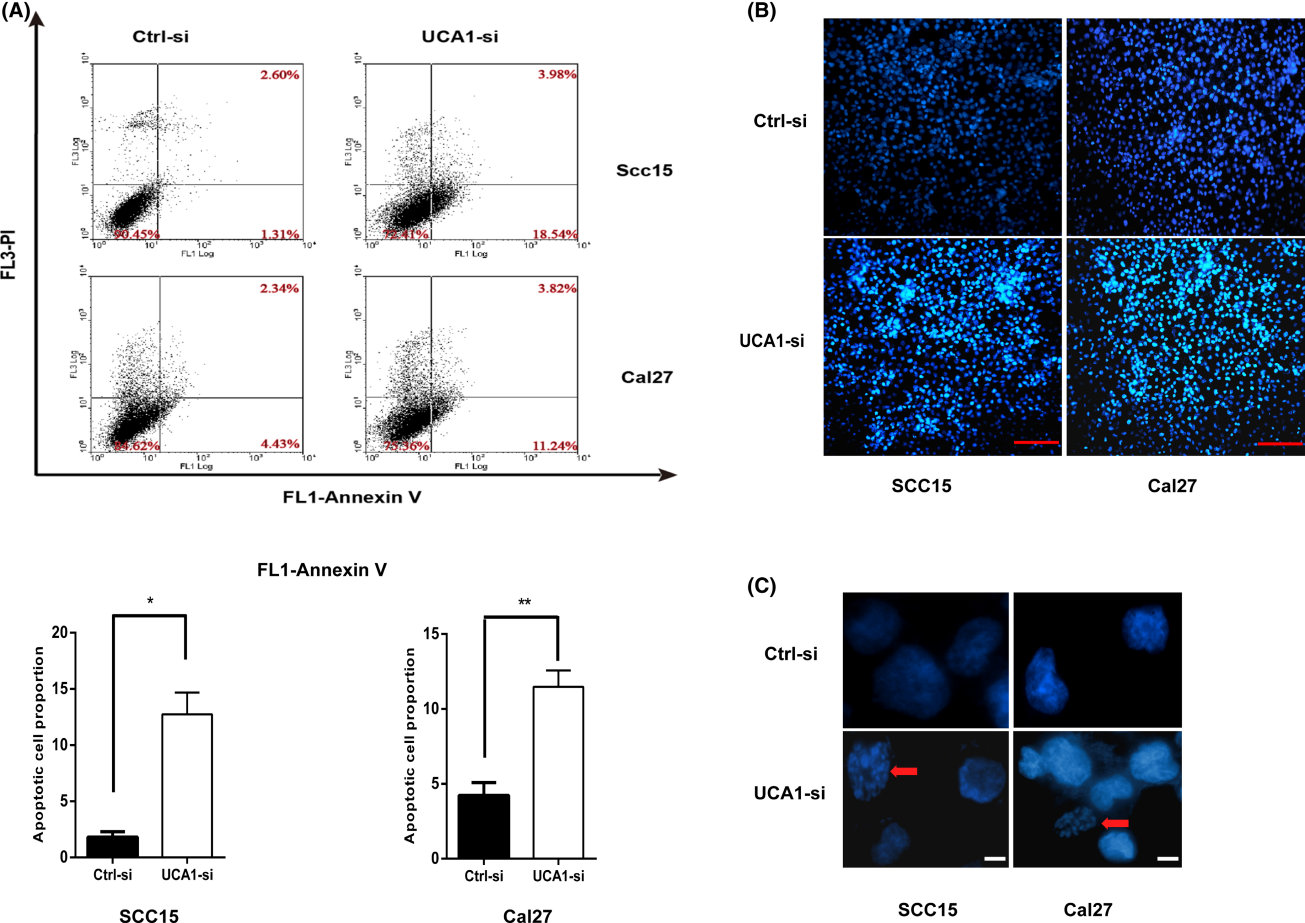


The revised Figure 5 is shown below:

**Figure 5:**
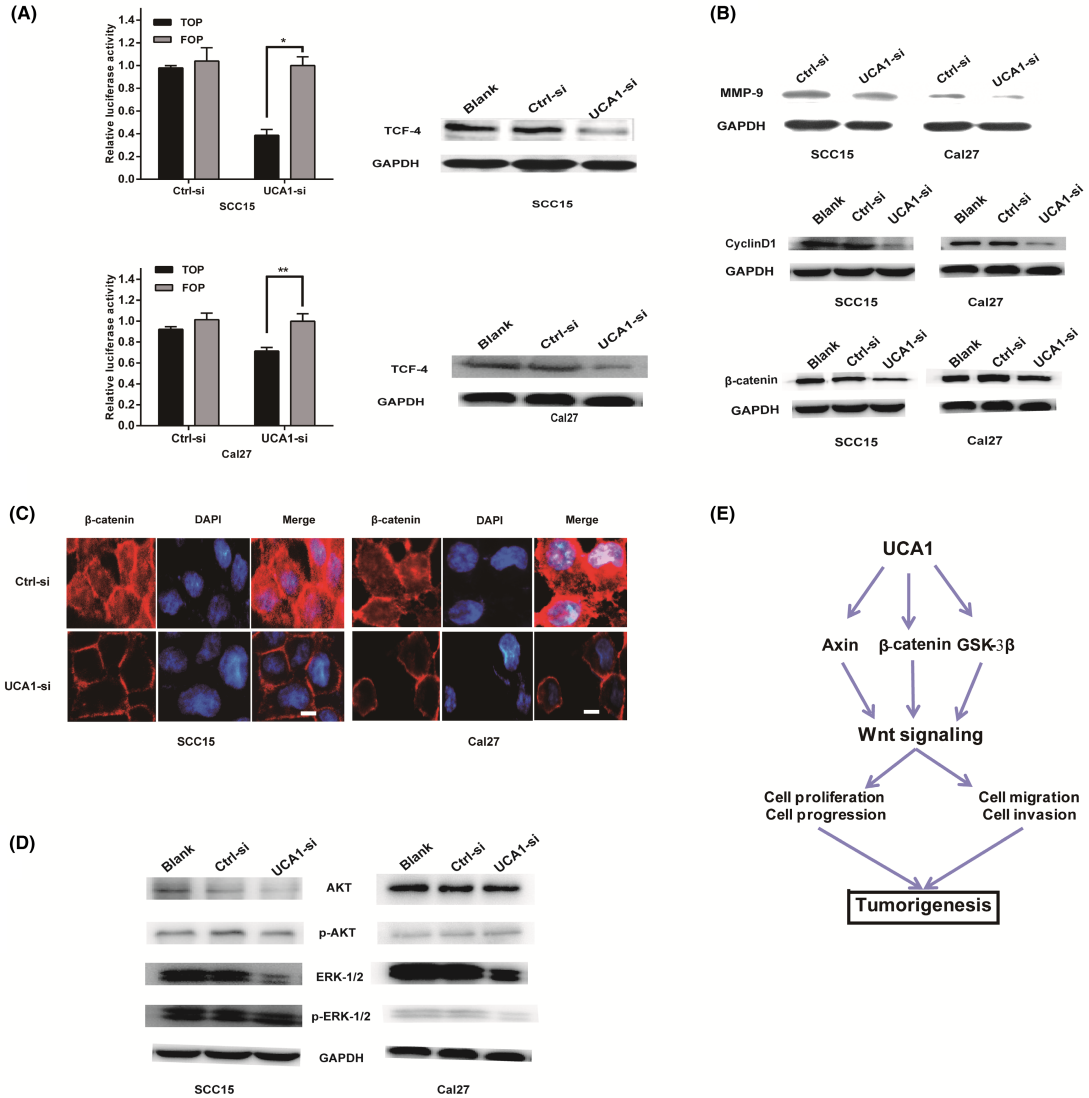
In the cell experiment, after transfection of cells in batches, protein extraction and freezing storage were concentrated for use in Western blot tests, subsequently. Therefore, several similar internal control bands were used in FIG. 5b and 5d. We add this information in the FIG. 5 legend as follow : “FIG 5. UCA1 promotes OSCC malignant progression through Wnt/ β‐catenin signaling pathway. (a) The luciferase reporter assay using TopFlash vectors was performed to detect β‐catenin TCF/LEF promoter activity, while Fopflash has mutated TCF binding sites, acting as a negative control. SCC15 and Cal27 cells were co‐transfected with different expression vectors as indicated. UCA1‐si treatment inhibited β‐catenin TCF/LEF promoter activity. (b) Western blot analysis of proteins (β‐catenin, TCF‐4) in the Wnt/β‐catenin signaling pathway and of downstream Wnt/β‐catenin signaling pathway targets, such as Cyclin D1, MMP‐9. (c) Immunofluorescence assay for β‐catenin implicates that the location of β‐catenin in cells moves from nuclear to cytoplasmic when the expression of UCA1 was silenced, and that β‐catenin expression decreased in the nucleus compared to Ctrl‐si.(d) Representative western blotting results for the protein expression levels of ERK1/2, p‐ERK1/2, AKT, p‐AKT from UCA1‐si or Ctrl‐si treated SCC15 and Cal27 cells. (e) Diagram depicting the regulation mechanism of UCA1 in the tumorigenesis of OSCC. (Data are presented as mean ± SD, *P < 0.05, **P < 0.01; GAPDH control was used multiple times and the same cell extract was used in the experiment conducted for (b) and (d).[Corrections made on 22 February 2023, after first online publication: The revised Figure 5 legend has been added in this version.]

The authors apologize for the errors.
